# Biomechanical Reconstruction Using the Tacit Learning System: Intuitive Control of Prosthetic Hand Rotation

**DOI:** 10.3389/fnbot.2016.00019

**Published:** 2016-11-29

**Authors:** Shintaro Oyama, Shingo Shimoda, Fady S. K. Alnajjar, Katsuyuki Iwatsuki, Minoru Hoshiyama, Hirotaka Tanaka, Hitoshi Hirata

**Affiliations:** ^1^Department of Hand Surgery, Nagoya University School of MedicineNagoya, Japan; ^2^Brain Science Institute-TOYOTA Collaboration Center, RIKENNagoya, Japan; ^3^Brain and Mind Research Center, Nagoya University School of MedicineNagoya, Japan; ^4^Department of Rehabilitation, Chubu Rosai HospitalNagoya, Japan

**Keywords:** myoelectric prosthesis, artificial intelligence, biomechanical function reconstruction, motor control, magnetoencephalography, interactive musculoskeletal modeling analysis, muscle, sensory synergy

## Abstract

**Background:** For mechanically reconstructing human biomechanical function, intuitive proportional control, and robustness to unexpected situations are required. Particularly, creating a functional hand prosthesis is a typical challenge in the reconstruction of lost biomechanical function. Nevertheless, currently available control algorithms are in the development phase. The most advanced algorithms for controlling multifunctional prosthesis are machine learning and pattern recognition of myoelectric signals. Despite the increase in computational speed, these methods cannot avoid the requirement of user consciousness and classified separation errors. “Tacit Learning System” is a simple but novel adaptive control strategy that can self-adapt its posture to environment changes. We introduced the strategy in the prosthesis rotation control to achieve compensatory reduction, as well as evaluated the system and its effects on the user.

**Methods:** We conducted a non-randomized study involving eight prosthesis users to perform a bar relocation task with/without Tacit Learning System support. Hand piece and body motions were recorded continuously with goniometers, videos, and a motion-capture system.

**Findings:** Reduction in the participants' upper extremity rotatory compensation motion was monitored during the relocation task in all participants. The estimated profile of total body energy consumption improved in five out of six participants.

**Interpretation:** Our system rapidly accomplished nearly natural motion without unexpected errors. The Tacit Learning System not only adapts human motions but also enhances the human ability to adapt to the system quickly, while the system amplifies compensation generated by the residual limb. The concept can be extended to various situations for reconstructing lost functions that can be compensated.

## Introduction

When we lose a functional part in our body (e.g., reaching out, walking, trunk control, breathing, watching, etc.), we not only lose functional output but also sensory feedback. Every biomechanical movement is a result of computations in the central-nervous-system (CNS) and at the same time, consecutive sensory feedback prediction and modification of motor behavior goes on in the cerebellum, allowing us to accomplish natural motion, and construct changes in response to the external environment (Brooks et al., 2015). Therefore, reconstruction of lost biomechanical function should not only include fine motor control but also dense sensory feedback that precisely, bi-directionally, and with high frequency communicates with the CNS. However, even the most advanced neuromotor reconstruction technology has not accomplished this communication, and lacks appropriate feedback for natural function. Furthermore, construction of a practical and ergonomic mechanical system that adapts to environmental changes within seconds is difficult due to lack of flexibility in current artificial machine learning.

One typical challenge of reconstructing lost function is the functional hand prosthesis. These are widely used in reconstruction on forearm amputees and congenital forearm deficient individuals for restoring their ability to reach and grasp. Among these, body power and myoelectric prostheses are widely used for motor control. In the past, body powered prosthesis were advantageous in cost, intuitiveness and sensory feedback, but not in function. Thus, a great effort was required to accomplish more function and natural movement in myoelectric prosthesis (Ciancio et al., 2016).

Developments in technology over the past few decades has improved control on multiple functions, with a primary focus on minimizing user burden and increasing prosthesis' function. Nevertheless, increasing the number of myoelectric input channels resulted in non-physiological muscle activation that required exhaustive training (Schulz et al., 2005). Target muscle re-innervation (Kuiken et al., 2007) may be one solution, but is too invasive and less beneficial for trans-radial amputees which represent the largest proportion of individuals with upper extremity deficiency (Hahne et al., 2012). The development of pattern recognition and machine learning techniques of electromyography (EMG) signals increased the number of degrees of freedom (DOFs) while keeping the number of utilized electrodes low. However, this technique has a critical limitation of low adaptability to environmental changes (Ciancio et al., 2016).

Meanwhile, a large number of studies have used the brain's plasticity to quickly adapt and reorganize cross-modal sensory integration for sensory feedback reconstruction. Since most of the work focuses on tactile feedback for adjusting grip force, it is still a challenge to reconstruct natural sensory feedback and mimic natural control. Recently, a few studies have reported increased sensory information density by neural implants (Ciancio et al., 2016); however, neurophysiological studies have indicated that position in space is estimated by integrating information from multiple sensory inputs rather than direct input. Moreover, as this integrated feedback is noise-robust, useful and cost-effective, adding appropriate sensory integration may result in better reconstruction (Alnajjar et al., 2015).

In our natural motion learning, we use two different modes, i.e., explicit and tacit learning. The former occurs with learner's awareness, while the latter takes place subliminally. When we perform a motor skill, there is a variety in the status of our neuromotor situation, which is subliminal and highly coordinated to express low dimensional motion. The key to a natural control strategy is management of this inherent redundancy in the musculoskeletal system mediated by a high number of DOFs with low dimensional outputs (Metzger et al., 2012).

Recently, several studies have shown that muscle synergy is like a neural strategy that the CNS has adopted to simplify the control of our redundant musculoskeletal system. Additionally, the importance of integrating environmental inputs into suitable low-dimensional signals before sending them to the CNS for simplified control have been documented (Alnajjar et al., 2015). Yet the neural dynamics inside the CNS have not been investigated in detail. Shimoda introduced a biological self-regulatory adaptive control strategy called “Tacit Learning System” (TLS) for posture control with self-sufficiency. This system is designed for unsupervised acquisition of skills or creation of new behavioral structures for adapting to environmental changes. Signal accumulation is a key factor for “Tacit Learning” in the adaptation process and primitive behaviors composed of several reflex actions are gradually tuned into suitable behaviors for the environment (Shimoda and Kimura, 2010; Shimoda et al., 2012, 2013).

Shimoda and his team have succeeded in controlling 36 DOFs in a humanoid bipedal locomotive robot using this TLS and demonstrated a wide adaptation capability to a redundant motor-skeletal system along with robustness to environmental changes compared to conventional machine learning algorithms (Shimoda et al., 2012). We thus hypothesized that introducing TLS into the biomechanical structure as a subsystem will integrate it with muscle synergy to control implicit motion with adaptation to environmental changes, allowing the user to concentrate on explicit tasks like grasping in myoelectric hand prosthesis. Clinically, a compensatory strategy to the rotation function of a lost wrist, involves using proximal residual limbs to achieve the necessary motion, result in increased burden on users that limit prosthesis usage (Metzger et al., 2012). This rotation function of reaching is an example of implicit motion. Consequently, we performed experiments to evaluate the efficacy of TLS in a prosthesis hand model, by appointing the system to regulate wrist rotation to minimize redundant compensatory motion as a biomimetic regulatory system while performing reaching tasks.

## Materials and methods

In this study, a non-randomized experiment was conducted to evaluate efficacy of the TLS and its effects on the central nervous system during the prosthesis control tasks.

### Prosthesis efficacy evaluation in bar relocation tasks

Seven men and one woman participated after giving informed consent. All participants were below elbow amputees, and experienced users of the conventional one-degree (hand open and close) myoelectric hand prosthesis. Table 1 shows the participants' demographic data.

**Table 1:** **Demographic data of participants**.

**No**.	**Age (y)**	**Sex**	**Side**	**Duration of myoelectric prosthesis use**	**Device type**
1	51	Male	Right	5 years	Ottobock8E44 = 6+10S17+10V38
2	40	Male	Right	8 years	Ottobock8E38 = 9
3	46	Female	Right	12 years	Ottobock8E38 = 6
4	41	Male	Right	6 months	Ottobock8E44 = 6+10S17+10V38
5	29	Male	Right	2 months	Ottobock8E44 = 6+10S17+10V38
6	52	Male	Right	3 years, 6 months	Ottobock8E38 = 6+10S17
7	33	Male	Left	1 year	Ottobock8E38 = 6+10S17+10V38
8	74	Male	Right	32 years	Ottobock8E38 = 6

**Figure 1:**
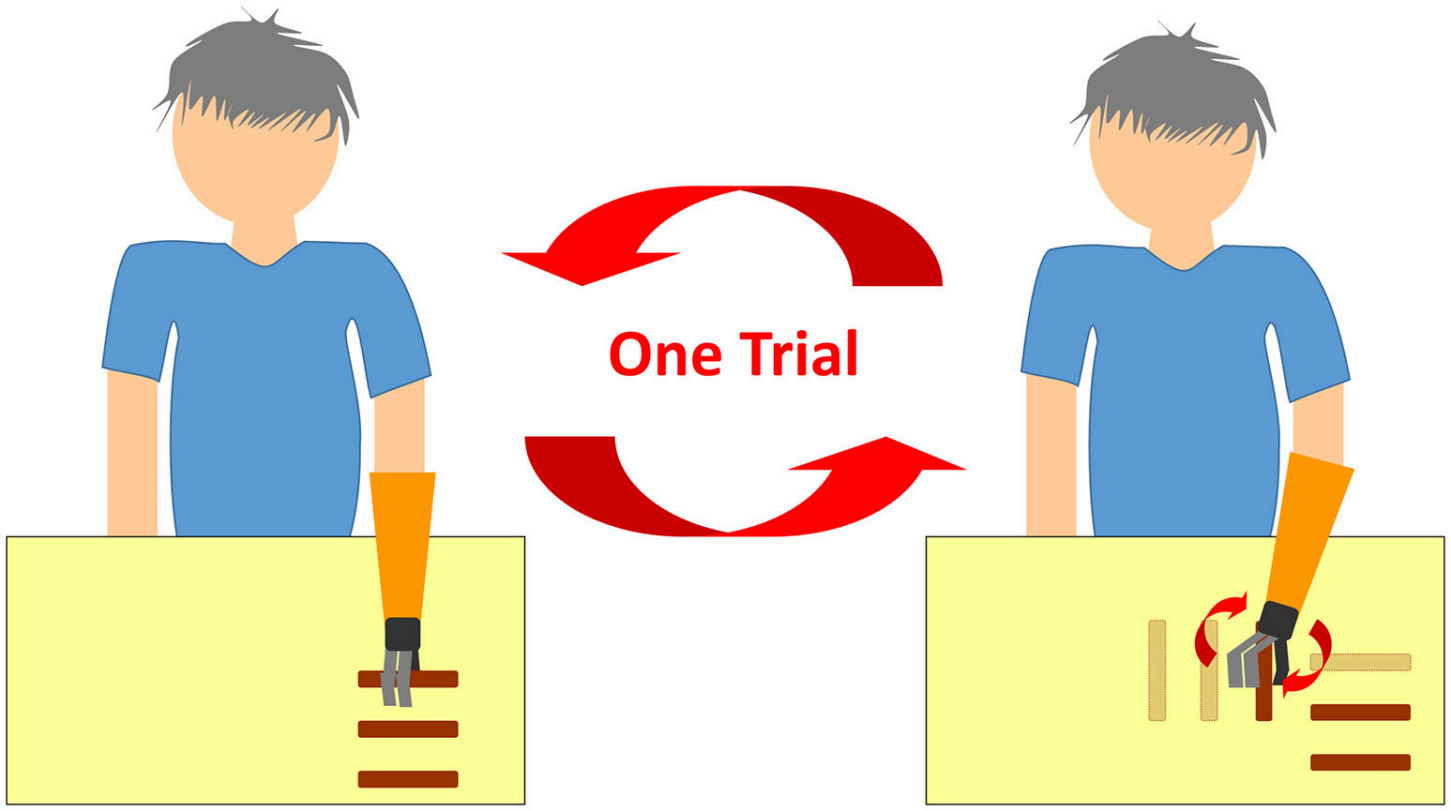
**A schematic figure of the trial**. After moving three bars vertically, the participants were instructed to place these three bars back to where they were horizontally.

Each participant's prosthesis handpiece was exchanged with the TLS handpiece and their remaining arm sockets were used in the trials. The open/close signal detector on the handpiece was connected to sensors in the socket to allow the participants to control hand motions as usual. Since we could not find adequate forearm rotation tasks for hand prosthesis in the past literature, we placed three plastic bars (3 cm diameter and 10 cm length with the central 3 cm part covered with Velcro tape to increase grasp) horizontally on a table. Participants sat in front of the table, reached out to hold the bars, placed them vertically and then back to horizontally three times (Figure 1). This exercise counted as one trial. Initialization of the system was performed as a pre-trial. Participants were instructed to stay still for 5 s with their shoulders at 0° flexion, rotation, and abduction, along with elbows at 0° flexion. Subsequently, they were instructed to repeat the trials until the rotational support of hand prosthesis made no more improvement. Twenty trials were done in approximately 10 min, and this was sufficient for every patient to achieve convergence of the parameters. Angles of shoulder joints derived from three goniometers placed on the participant's shoulder (flexion, rotation, and abduction) were monitored and fed back to the TLS. Participants' movements were recorded with a computer vision based human body motion capture tracking system (Section Tacit Learning Handpiece and Data Preprocessing) First person sight video (FPV) (Video 1, 2) recording was performed by the camera (HERO, GoPro, Inc., CA, USA) attached to the prosthesis socket. We conducted descriptive type questionnaires to determine participant satisfaction and how effective the participants felt the system was.

### Tacit learning handpiece and data preprocessing

**Figure 2:**
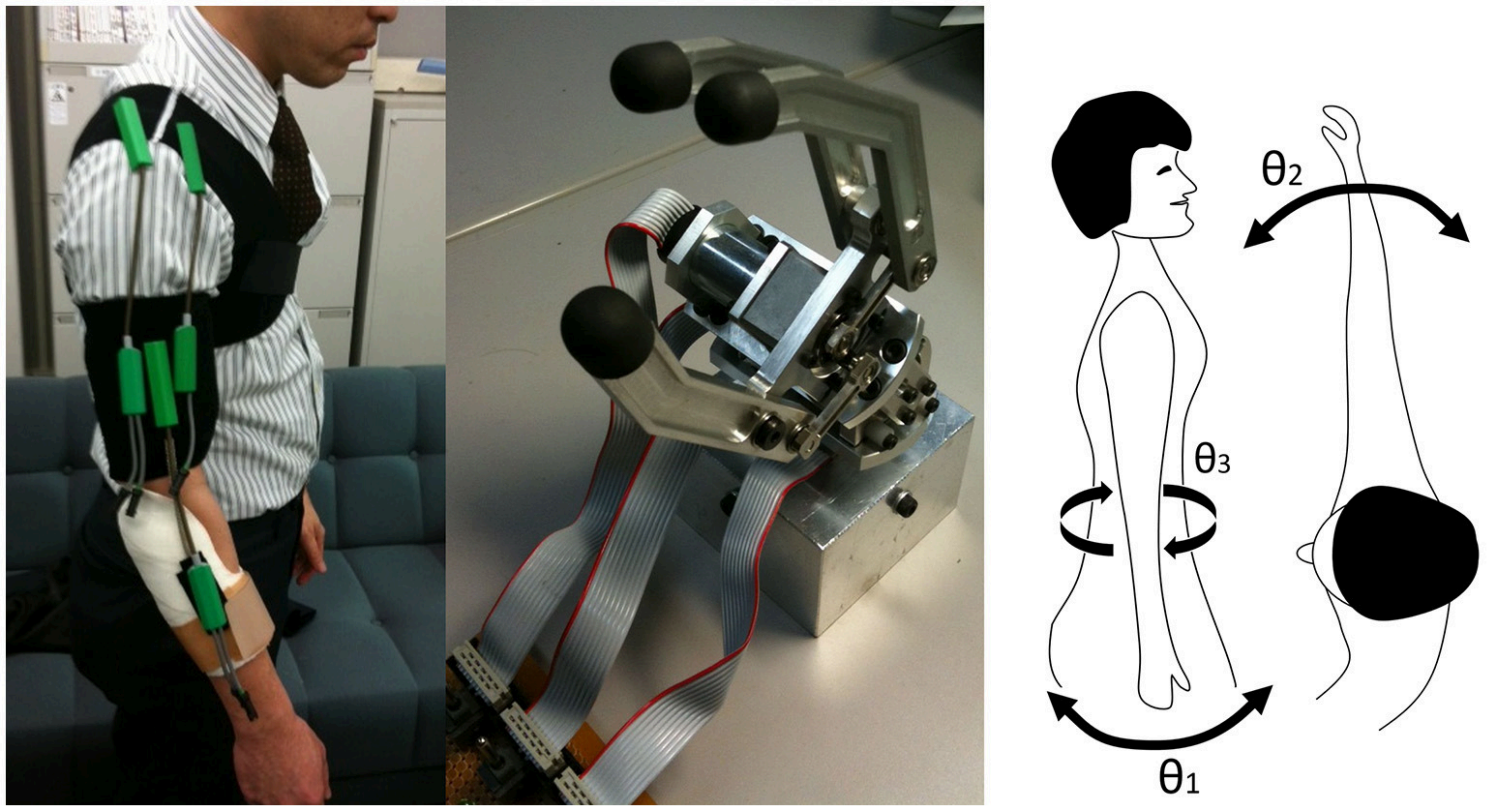
**Three goniometers were attached as shown (left) and linked to the handpiece (center) for measuring the upper extremity joint angle (θ_1_, θ_2_, θ_3_, right)**.

The system consisted of three goniometer sensors to measure angles of shoulder flexion (θ_1_), horizontal flexion (θ_2_), rotation (θ_3_), and a handpiece with two actuators (rotation and grip) (Figure 2). One actuator was for handpiece wrist rotation. Rotation angle θ_*r*_ was a desired angle of prosthesis wrist rotation, controlled by a low-level controller embedded in the hardware. The other actuator was for grip with an on-off control provided by surface EMG sensing which is commonly used by commercial prosthesis. When the shoulder angles exceeded pre-defined threshold values θ_t_ (the value found at unnatural postures), the system tuned the control gain, accumulating extremity joint angles. The control and adaptation laws were defined as follows:

(1) $$\theta_{r}=k\Theta -{\dot{\theta }}_{r}$$

(2) k=∫ qdt

(3) $$q=\{\begin{array}{c} \Theta \;|\Theta |\geq \theta_{t}\\ 0\;\;|\Theta |<\theta_{t}\; \end{array}$$

(4) $$\Theta =k_{1}\theta_{1}+k_{2}\theta_{2}+k_{3}\theta_{3}$$

When a linear combination of residual upper limb joint angles Θ in Expression (4) exceeded the settled threshold angle θ_*t*_ in Expression (3), primary reflex modulated rotatory assistance angle θ_*r*_ depending on Θ in Expressions (1) and (2). Expression (1) was a speed control component of rotation. A previous mathematical study suggests that biological arm kinematics are optimized by total energy expenditure (Berret et al., 2008), which is positively correlated to the total joint angle Θ. Thus, we determined the control law of system as minimization of Θ. In this experiment, we set θ_*t*_ = 1, *k*_1_ = 0.1, *k*_2_ = 0.1, and *k*_3_ = 0.5 as the initial values.

### Motion capture system

Kinematic patterns of the participants' movements were captured with a motion capture system (Workstation 5.2.4, VICON). Twenty-four markers (spheres covered with reflective tape) were attached to various parts of the participant's body and prosthesis prior to the experiment. The motion capture system consisted of six cameras, which tracked and reconstructed the motion of each of the recorded markers in 3D space.

### Data analysis

We focused on the tacit learning rotational control of prosthesis on this study.

Hence, we computed system energy consumption by using the software for Interactive Musculoskeletal Modeling (SIMM, MusculoGraphics, Inc., Santa Rosa, California, USA). It is a graphical software system for developing and analyzing models of musculoskeletal structures, and performs inverse dynamics calculations from motion capture data (Delp and Loan, 1995; Neptune et al., 2008). It creates a musculoskeletal model consisting of representations of bones, muscles, and ligaments by calculating the joint moments. In this study, we used a standardized musculoskeletal model calculated from the participants' body weight, height, and sex. Pre-trial system energy in all participants was normalized as one.

## Results

**Figure 3:**
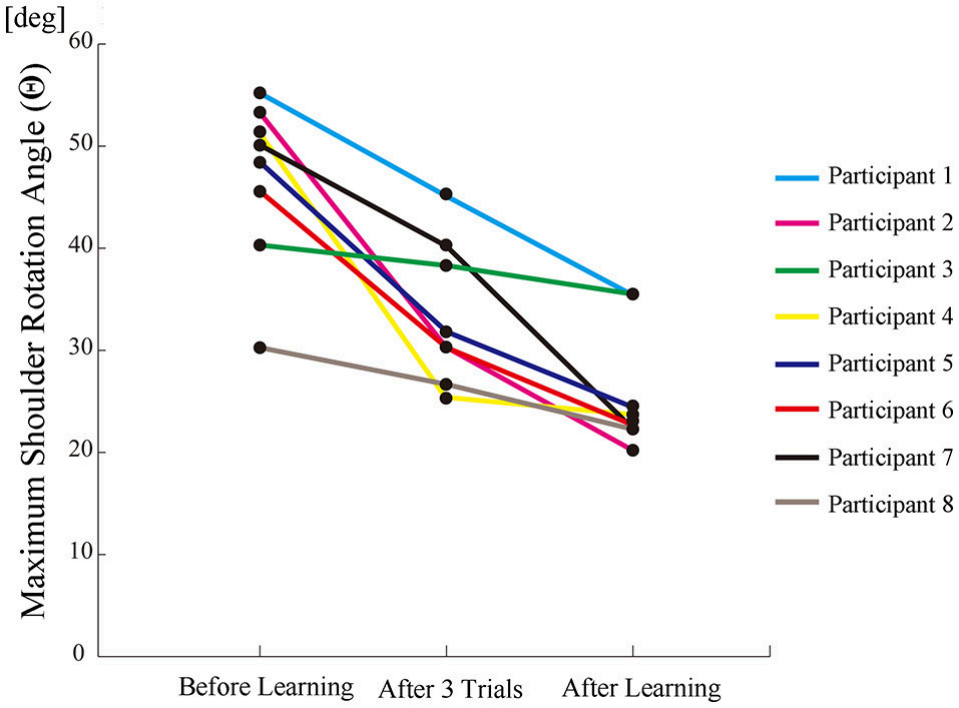
**The maximum shoulder rotation angle (as compensation movement for forearm rotation tasks) decreased after trials**.

**Figure 4:**
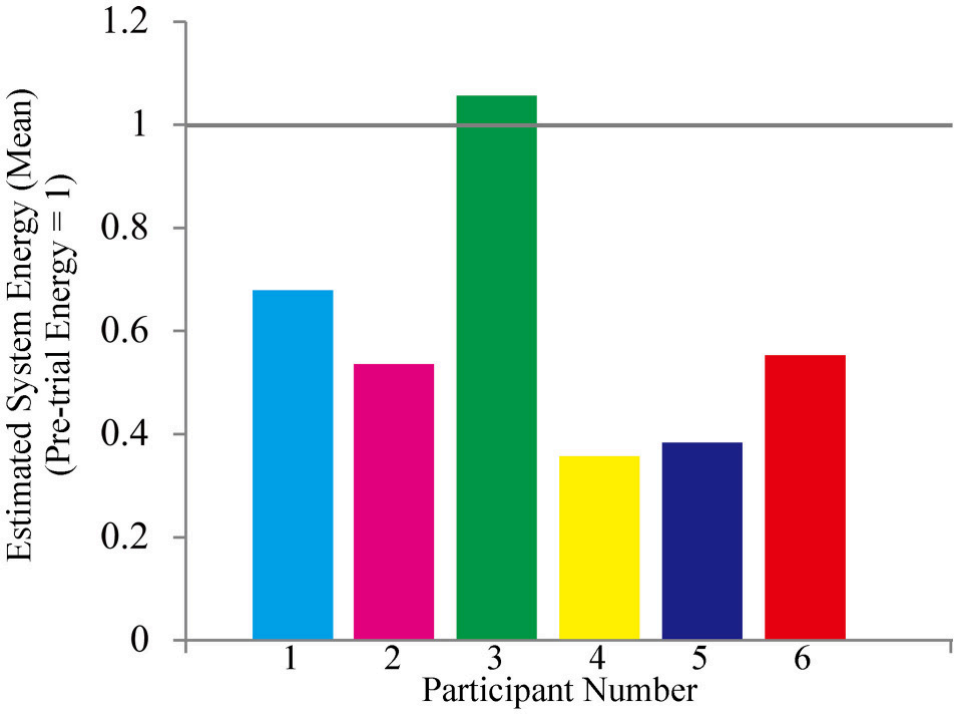
**Estimated mean system energy decreased significantly in five out of six participants**.

**Figure 5:**
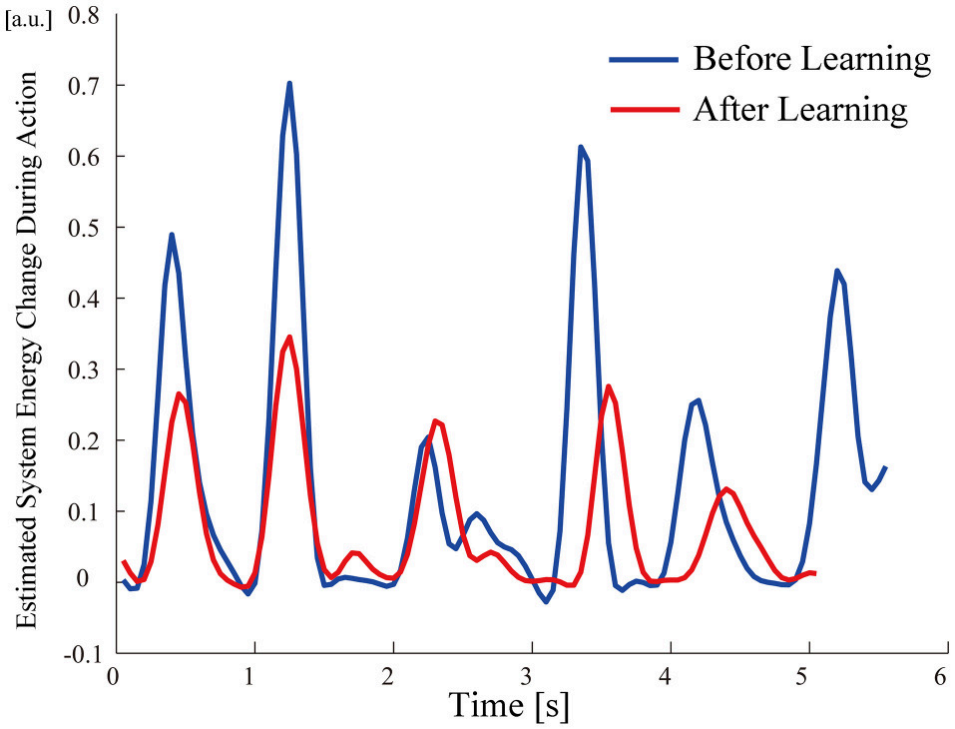
**Estimated system energy change during trials before/after TLS learning in participant 1**.

**Figure 6:**
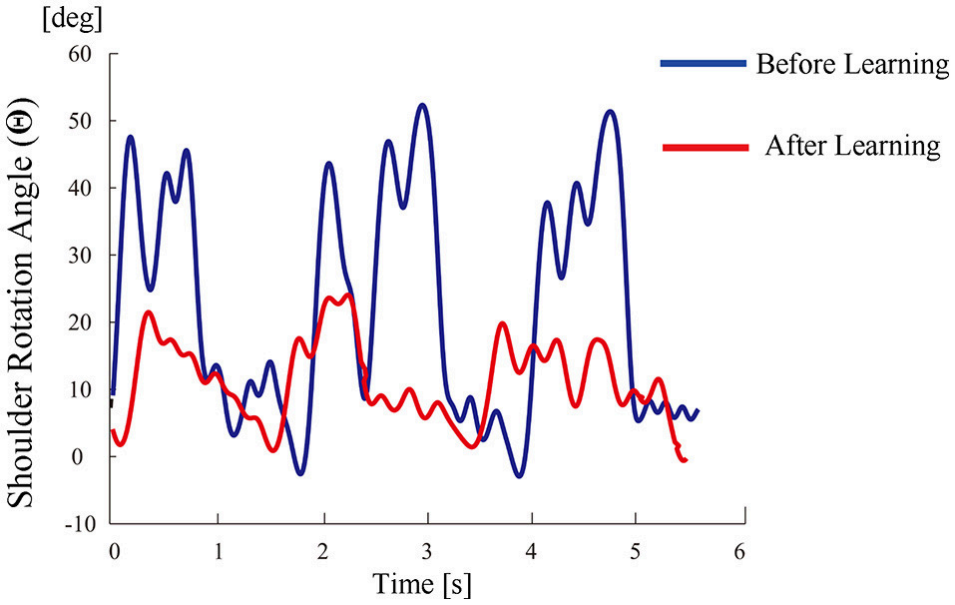
**Shoulder rotation angle (as compensation movement for forearm rotation tasks) during tria/FCls before/after TLS learning in participant 1**.

**Figure 7:**
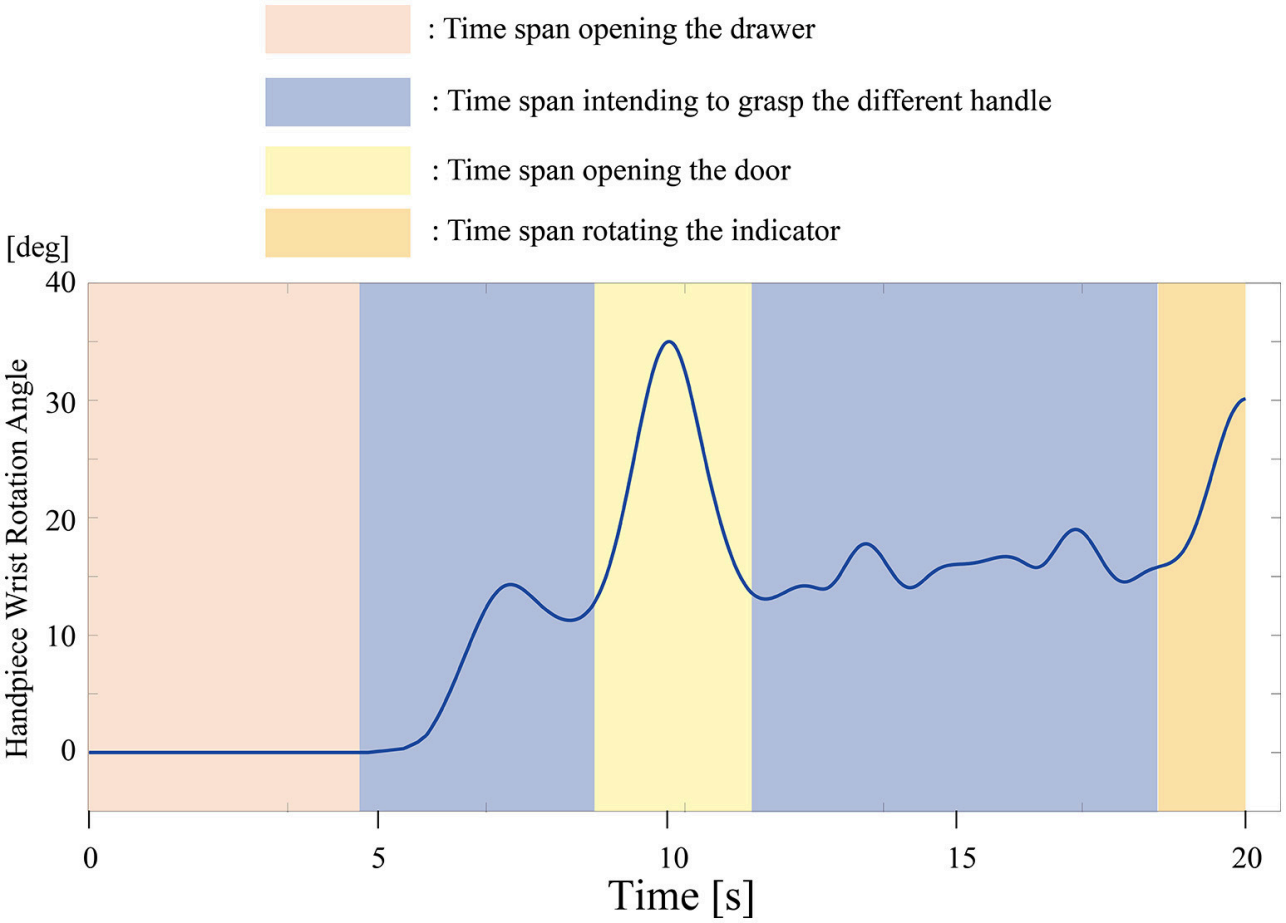
**Wrist rotation angle of the prosthesis during the drawer and indicator tasks**. Rotational support was efficient, even though tacit learning TLS did not experience the tasks.

All participants successfully completed their assigned tasks. Online video (Video 1, 2, 3) shows participant 3 working on his tasks. “After learning” represents 20 trials after the first one. Adaptation advanced in both wearer and prosthesis in a short while as shown in the videos (Video 1: Without TLS assistance. Video 2: After twenty trials). After 20 trials, the shoulder rotation angle (θ_3_) decreased in all participants as shown in Figure 3. Total system energy estimated by SIMM decreased in five out of six patients (Figure 4). Energy estimation was not possible in participants 7 and 8 due to failure of the motion-capture marker. Figure 5 shows changes in the actual estimated system energy data during trials in participant 1. The graphs show system energy before and after TLS learning. The compensation rotation angle of shoulder [Θ in Section Tacit Learning Handpiece and Data Preprocessing, Expression (4)] in participant 1 decreased after TLS learning as shown in Figure 6. Seven out of eight participants were comfortable with TLS assistance. No participant required special training before the trials. After TLS learning of bar rotational tasks, participant 8 volunteered to open two types of drawers and turn the oven indicator (Video 4). Figure 7 shows rotation angle of the prosthesis wrist during the tasks. Although TLS did not experience any of these tasks, it provided good assistance and showed generalized performance for rotational support despite changes in arm posture. This participant was also satisfied with the intuitiveness of TLS support as determined by the descriptive type questionnaires.

## Discussion

A good hand prosthesis should reconstruct the original dexterity of human hands. While far from complete, in this endeavor we replicated one of the most complex biomechanical structures. Improvements in EMG signal analysis (Tenore et al., 2009), Targeted Muscular Reinnervation (TMR) including sensory feedback (Kuiken et al., 2007; Ohnishi et al., 2007; Li et al., 2010), brain interface (Yanagisawa et al., 2011), peripheral nerve interface (Navarro et al., 2005), and new training systems (Pilarski et al., 2011) were invented, but these methods required a certain period of special training or special surgery invasions. Furthermore, none of these methods satisfied the contrary demands for intuitiveness, multi-functionality, and cost. Moreover, due to the lack of flexibility in present control methods to adapt with environmental changes including complex nature of the bio-signals, repeated calibration is often required by patients and physiotherapists (Ciancio et al., 2016).

The present work focuses on reconstructing each joint's movement, but not muscle synergy. Reconstruction of muscle synergy does not involve isolating and reorganizing bio-kinematic outputs from residual function, but expanding muscle synergy in a biological way. In other words, an optimization algorithm should be introduced for mimicking human-like motion and finding the natural output from residual limb to cope with. Shimoda described that human kinematic output is unpredictable for machines with a model-based strategy that does not represent certain posture situations (i.e., forearm rotations, elbow flexion/extension) due to intrinsic fragility (Shimoda and Kimura, 2008). To cope with all motions, it is necessary to model all possible posture changes and device control actions in every model. To solve these issues, various types of bio-mimetic and self-organizing learning methods including artificial neural networks have been proposed, but the capability of current learning methods to adapt to unknown situations is not sufficient in terms of learning speed and the level of generalization.

The “Tacit Learning System” introduced by Shimoda has two main advantages for controlling the prosthesis compared to other control methods: learning speed and a simple, inexpensive system with intrinsic robustness (Shimoda et al., 2012, 2013). Furthermore, as Alnajjar described, this controller has a role in reduction of sensory stimulus dimension. This is called “sensory synergy” in contrast to muscle synergy. They defined “sensory synergy” as “a group of weighted sensory inputs whose function is to provide the quality of the resulting motion as feedback to the CNS through a single synergy recruitment signal in order to facilitate the generation of the next command, thus accelerating the search time for the optimal muscle synergy.” In particular, in TLS, the controller modulates sensory synergies contributed by acquired sensory signals and inferred artificial sensory synergies into motor commands. Consequently, activated motor commands of the prosthesis enable intuitive motor control by the wearer and simultaneous confirmation with visual feedback. In short, the output of sensory synergy is used as an input to both the CNS and the TLS, and control signals for the prosthesis device are created through motor synergy that combines signals from the CNS and the prosthesis device (Alnajjar et al., 2015).

Our results from the bar relocation experiment convinced us that this system has high affinity toward the CNS. It was easy to add on the conventional system, required no special training, reduced users' burden and is low-cost. The level of satisfaction was high.

Recently, we reported a case report from a magnetoencephalography study on the effect of the TLS system on CNS. This report showed that the coherence value among sensorimotor-related cortices in the dominant hemisphere increased only while watching a video of oneself using the prosthesis with TLS support and vice versa. This result is no more than a showcase, but we are preparing for a future clinical study evaluating the effect of the “Tacit Learning System” prosthesis on CNS based on the evidence of this basic study.

A limitation of this study is that we tested this system on limited tasks and thus, it is still in the prototype phase currently. We tried several learning motions and determined that various motions could progress the learning in a similar way to that shown in the results. This robustness is justified with the experiments of motion generalizations by the drawer opening task. In cases where less extreme motions were used in the training sessions, the learning speed was slow, and it took many trials to learn the appropriate behaviors. For this study, we choose a simple relocation task to control the learning environment for all participants and to compare the differences in the learning process. The users did not try the system in real life tasks like cooking, housework etc. However, results of additional tasks performed by participant 8 in an additional experiment suggest robustness of the system in different situations. Short battery life is also a concern. The system continuously senses upper limb motions and tries to adjust prosthesis positions at all times, so battery drainage is three to five times greater than the conventional systems. Setting the threshold adjustment may be a solution. Higher threshold to a TLS support may increase battery life but may result in reduced support, which needs to be considered according to the users' lifestyle.

In summary, we introduced a novel “Tacit Learning System,” a self-regulatory strategy in a myoelectric prosthesis, to control wrist rotation and confirmed its efficacy in conventional type myoelectric prosthesis users. We infer that TLS showed the ability to recover the lost function by adjusting compensatory overreaction generated by residual function. Theoretically, it can be used for recovering functions in other situations such as lower limb amputation, palsy in association with functional electric stimulation, or even in ventilation failure if residual function is present.

## Human and animal rights and informed consent

This study was approved by the Ethics Committee of the Nagoya University, School of Medicine (Approval number 2012-0145). All participants gave informed consent before enrolling in the study, and all procedures were performed in accordance with ethical standards of the responsible committee on human experimentation and with the Helsinki Declaration of 1975, revised in 2000 and 2008.

## Author contributions

Conceived and designed the experiment: SO, SS, KI, and HH. Performed and analyzed the data for experiment: SO, SS, FA, and KI. “Tacit Learning System” design, settings, and construction: SS and FA. Participant registrations: KI and HT. Wrote the paper: SO, SS, KI, MH, and HH.

### Conflict of interest statement

The authors declare that the research was conducted in the absence of any commercial or financial relationships that could be construed as a potential conflict of interest.
